# Glibenclamide Serves as a Potent Vasopressor to Treat Vasoplegia After Cardiopulmonary Bypass and Reperfusion in a Porcine Model

**DOI:** 10.3390/ijms26094040

**Published:** 2025-04-24

**Authors:** Andreas Winter, Pascal Nepper, Marcus Hermann, Franziska Bayer, Stephanie Riess, Razan Salem, Jan Hlavicka, Anatol Prinzing, Florian Hecker, Tomas Holubec, Kai Zacharowski, Thomas Walther, Fabian Emrich

**Affiliations:** 1 Department of Cardiovascular Surgery, University Hospital Frankfurt, Goethe University, 60629 Frankfurt am Main, Germanymarcus.hermann@life-systems.de (M.H.); jan.hlavicka@unimedizin-ffm.de (J.H.); tomasholubec@email.cz (T.H.); thomas.walther@unimedizin-ffm.de (T.W.); fabian.emrich@t-online.de (F.E.); 2 Central Research Unit, Goethe University Frankfurt, 60629 Frankfurt am Main, Germany; franziska.bayer@unimedizin-ffm.de; 3 Department of Anesthesiology, Intensive Care Medicine and Pain Therapy, University Hospital Frankfurt, Goethe University, 60629 Frankfurt am Main, Germany; 4 Department of Cardiovascular and Thoracic Surgery, University Hospital Augsburg, 86156 Augsburg, Germany

**Keywords:** cardiopulmonary bypass, reperfusion, Glibenclamide

## Abstract

The hemodynamic stabilization of patients after complex cardiac surgery is a daily challenge. The use of high doses of catecholamines is common but has potential adverse effects. Glibenclamide, a K_ATP_ blocker, seems to attenuate vasoplegia in different animal models of septic shock. Therefore, the aim of this study was to investigate the impact of Glibenclamide on the vasoplegic syndrome after cardiopulmonary bypass in a porcine model. In this experimental study, 20 landrace pigs were randomized into two groups and examined: In the control group, standard medical therapy, including norepinephrine, was used, and in the study group standard medical therapy plus additional Glibenclamide was administered. Following general anesthesia, prolonged cardiopulmonary bypass and aortic cross-clamping was performed. In the study group, Glibenclamide was administered 45 min after weaning from cardiopulmonary bypass. The dosage used was 10 mg/kg as a bolus, followed by a continuous infusion of 10 mg/kg/h. Hemodynamic and laboratory measurements were performed. Glibenclamide had a relevant effect on circulatory parameters. With increasing vascular resistance and blood pressure, norepinephrine was able to be reduced. While the heart rate dropped to physiological levels, the cardiac index decreased as well. The results lead to the conclusion that Glibenclamide was able to break through vasoplegic syndrome and could therefore serve as a potent drug to stabilize patients after cardiac surgery.

## 1. Introduction

Despite the frequent use of minimally invasive approaches, conventional cardiopulmonary bypass (CPB) and cardiac arrest remain routine techniques in complex cardiac surgical procedures.

CPB is often associated with an inflammatory response during reperfusion, vasoplegic syndrome (VS), and consecutively the need for high doses of vasopressors during and after the procedure. VS leads to increased morbidity and mortality of the patients. It is characterized by vasodilatation and reduced systemic vascular resistance (SVRI), leading to a distributive shock with low blood pressure and impaired peripheral perfusion [1].

VS is reported to occur in up to 20% to 63% of patients [2], and is related to longer CBP duration [3]. The pathogenesis is considered to be multifactorial. Contact activation, adenosine triphosphate (ATP) deficiency, activation of the complement and coagulation systems, as well as various anesthetics and their effects on the vasculature are potential causes [1,2,4,5,6,7].

Another mechanism associated with the development of pathological vasodilation during reperfusion is the activation of ATP-sensitive potassium channels (K_ATP_). With a reduced concentration of ATP in the case of ischemic reperfusion, potassium channels are no longer inhibited and are therefore passively activated. This leads to relaxation of the smooth vascular muscles of peripheral arteries [8].

This study aims to evaluate a new medical approach to avoid and reduce VS in a porcine model with prolonged CPB. A sulfonylurea called Glibenclamide, which is already approved as an oral antidiabetic agent and currently investigated in a phase II study as an intravenous agent against cerebral edema [9,10,11,12], inhibits this K_ATP_ and could therefore attenuate the VS [13,14]. In our study, we used a dosage of 10 mg/kg as a bolus infusion, followed by a continuous administration of 10 mg/kg/h until the end of the experiment.

The aim of this study was to investigate the impact of the intravenous application of the sulfonylurea Glibenclamide on VS after CPB in a porcine model.

## 2. Results

### 2.1. Hemodynamic Measurements

#### 2.1.1. Control Group (CG)

**Figure 1:**
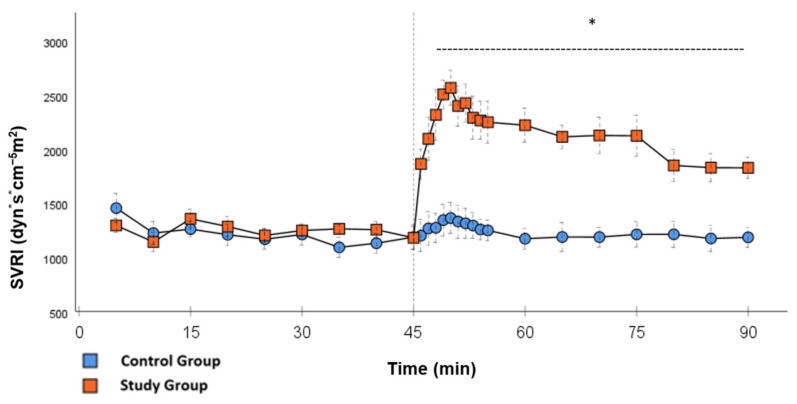
**SVRI:** Continuous monitoring of the systemic vascular resistance index during post-CPB observation. Values are expressed as mean ± SEM. * (*p* < 0.05) represents significant differences between the study and control groups at the respective time steps (Mann–Whitney *U* test). The dashed line displays the timeframe of significance. The reference line at 45 min signals the start of Glibenclamide application.

**Figure 2:**
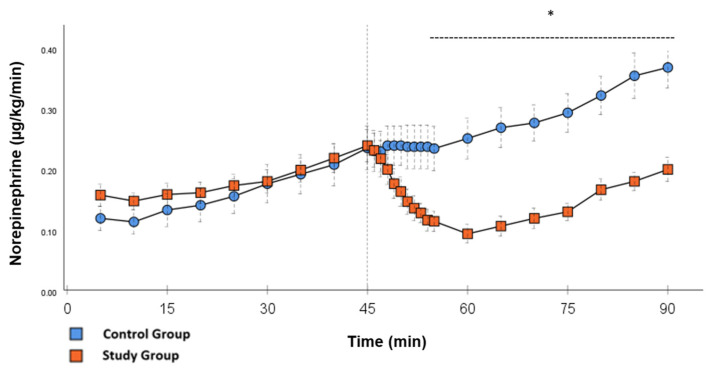
**Norepinephrine:** Continuous application of norepinephrine during post-CPB observation. Values are expressed as mean ± SEM. * (*p* < 0.05) represents significant differences between the study and control groups at the respective time steps (Mann–Whitney *U* test). The dashed line displays the timeframe of significance. The reference line at 45 min signals the start of Glibenclamide application.

**Figure 3:**
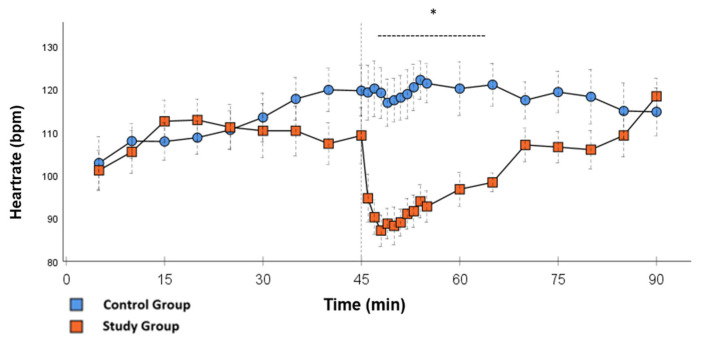
**Heartrate:** Continuous monitoring of the heart rate during post-CPB observation. Values are expressed as mean ± SEM. * (*p* < 0.05) represents significant differences between the study and control groups at the respective time stamps (Independent samples *t*-test). The dashed line displays the time frame of significance. The reference line at 45 min signals the start of Glibenclamide application.

In the CG, VS occurred in all animals during post-CPB observation. This could be seen in the following hemodynamical changes: Mean arterial pressure (MAP) remained constantly low with the need for high-dose norepinephrine therapy. SVRI dropped significantly during post-CPB observation, from 1459 ± 135.7 dyn·sec·cm^−5^·m^2^ to 1187.5 ± 93.7 dyn·sec·cm^−5^·m^2^ at the end of the experiment (Figure 1; *p* = 0.022). In order to keep the MAP between 60 and 70 mmHg, the norepinephrine doses had to be adjusted from 0.12 ± 0.02 µg/kg/min starting post-CPB observation up to 0.373 ± 0.035 µg/kg/min at the end of the experiment (Figure 2; *p* = 0.005). The heart rate (HR) raised from 102.8 ± 6.1 bpm min starting post-CPB observation to 119.6 ± 5.8 bpm at 45 min (Figure 3; *p* = 0.008) and stayed at this level until the end of the experiment. There were no significant changes in Global end-diastolic volume index, Extravascular lung water index, Global ejection fraction, Cardiac index (CI), and Central venous pressure (CVP) during the post-CPB observations.

#### 2.1.2. Study Group (SG) vs. Control Group (CG)

**Figure 4:**
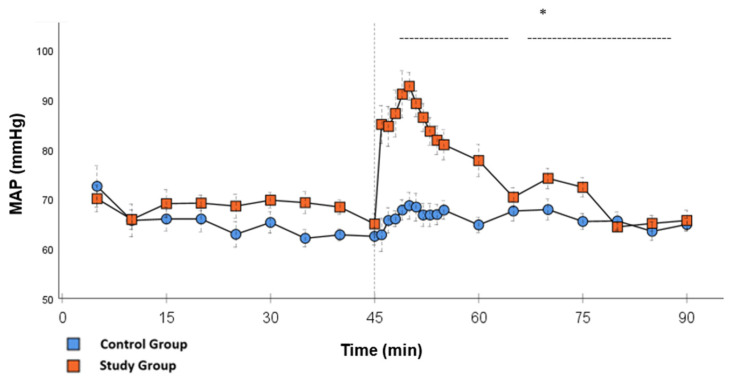
**MAP:** Continuous monitoring of the mean arterial pressure during post-CPB observation. Values are expressed as mean ± SEM. * (*p* < 0.05) represents significant differences between the study and control groups at the respective time steps (Mann–Whitney *U* Test). The dashed line displays the timeframe of significance. The reference line at 45 min signals the start of Glibenclamide application.

**Figure 5:**
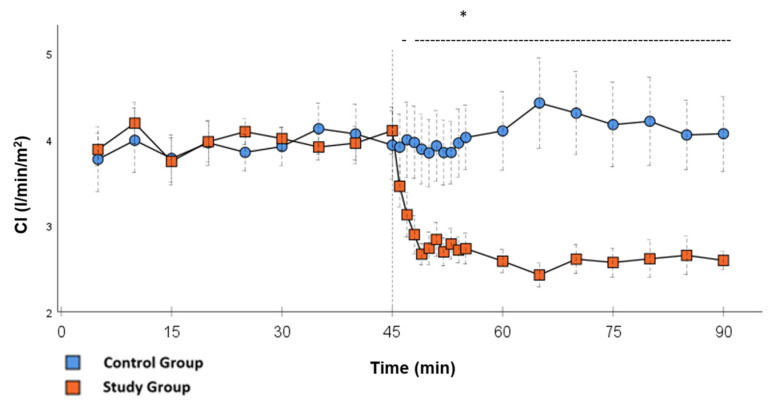
**CI:** Continuous monitoring of the pulse contour cardiac index during post-CPB observation. Values are expressed as mean ± SEM. * (*p* < 0.05) represents significant differences between the study and control groups at the respective time steps (Mann–Whitney *U* test). The dashed line displays the timeframe of significance. The reference line at 45 min signals the start of Glibenclamide application.

Significant hemodynamic differences between the groups were seen in MAP, SVRI, HR, CI, and lactate. After the application of Glibenclamide at 45 min post-CPB, the HR dropped significantly from 109.2 ± 6.5 bpm to 87.1 ± 3.6 bpm within 3 min in the SG compared to CG 119.1 ± 5.9 bpm (Figure 3; *p* < 0.001). Simultaneously, MAP increased from 65 ± 2.1 mmHg to 92.8 ± 2.9 mmHg in 5 min at 50 min post-CPB compared to CG 68.7 ± 2.7 mmHg (Figure 4; *p* < 0.001). The necessary norepinephrine dose could be reduced from 0.242 ± 0.027 µg/kg/min at 45 min post-CPB to 0.094 ± 0.016 µg/kg/min 15 min after application at 60 min post-CPB compared to CG 0.254 ± 0.035 µg/kg/min at 60 min post-CPB (Figure 2; *p* < 0.001). The SVRI increased from 1182.8 ± 103.8 dyn·sec·cm^−5^·m^2^ to 2576.9 ± 164.2 dyn·sec·cm^−5^·m^2^ in 5 min at 50 min post-CPB compared to CG 1366.6 ± 143 dyn·sec·cm^−5^·m^2^ at 50 min post-CPB (Figure 1; *p* < 0.001). The SVRI stayed significantly higher compared to the CG until the end of the experiment. CI dropped from 4.1 ± 0.3 L/min/m^2^ to 2.7 ± 0.13 L/min/m^2^ within 4 min at 49 min post-CPB compared to CG 3.9 ± 0.41 L/min/m^2^ at 49 min post-CPB (Figure 5; *p* = 0.004). While the effects of Glibenclamide on HR and MAP were only transient, the effect on CI and SVRI lasted until the end of the experiment. No further significant hemodynamic differences were observed.

### 2.2. Laboratory Testing

**Figure 6:**
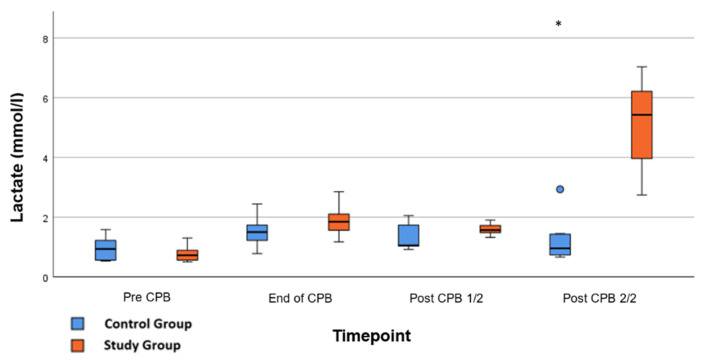
**Lactate:** Concentration of serum-lactate during whole experiment. Pre-CPB is before surgery, End of CPB is at the end of cardiopulmonary bypass, Post CPB 1/2 is just before the application of Glibenclamide in the study group, and Post CPB 2/2 represents the end of the experiment. Values are expressed as boxplots, continuing the median as a horizontal line inside the box. The first and third quantiles are the edges of the box. Whiskers have a distance of about 1.5 interquartile range to the edges. Outliers are plotted as dots. * (*p* < 0.05) represents significant differences between the Glibenclamide and control group at the respective time steps (Mann–Whitney *U* test).

Significant effects were seen in the lactate concentration after application of Glibenclamide; serum-lactate raised from 1.6 ± 0.6 mmol/L to 5.2 ± 0.4 mmol/L at the end of the experiment, 90 min post-CPB compared to CG 1.219 ± 0.2 mmol/L (Figure 6; *p* < 0.001). No further significant differences in the laboratory testing or the ELISAs were found. In particular, there was no significant difference in plasma glucose levels between CG and CG at the end of the experiment (*p* = 0.766).

## 3. Discussion

VS with loss of vascular resistance is a frequently observed complication after cardiac surgery with prolonged cardiopulmonary bypass [15,16,17]. For hemodynamic stabilization, high doses of norepinephrine are then often required during reperfusion after aortic cross-clamping. To prevent high doses of vasopressors and their adverse effects, we were able to make the following observations:

The application of Glibenclamide had significant effects on the hemodynamic parameters. These effects were either short- (MAP and HR) or long-lasting (SVRI, CI, Norepinephrine). By increasing MAP within a short time, it was possible to reduce the norepinephrine dose to a minimum. Multiple studies used 10 mg/kg Glibenclamide as a bolus injection dose with the same continuous dose over time [14,18]. This experience was used to visualize the effect of the study drug as clearly as possible. The T_1/2_ of Glibenclamide is described to be around 120 min [19]. Therefore, the effect of the drug can be monitored through the study design. In the setting of cardiovascular surgery, it is necessary to find the optimal dose of the bolus and continuous infusion to prevent hypertensive episodes shortly after surgery and to ensure the drug has a long-lasting effect. Norepinephrine doses stayed significantly lower until the end of the experiment compared to the CG, which could be the effect of continuous application. After the initial bolus administration, another effect of the continuous infusion was the significantly higher values of SVRI, not only in the first 15 min but also until the end of the experiment. The clear increase in blood pressure can be explained by an increase in vascular tension and thus afterload, which is represented by the SVRI. In contrast, in the CG, higher levels of norepinephrine were not able to generate a similar SVRI over the same period of time. This suggests that Glibenclamide has an α-1receptor independent effect on the vascular tone. In addition, synergistic effects of Glibenclamide and norepinephrine are known from a study in a porcine model of hemorrhagic shock. In this study, Glibenclamide resulted in an improved response of the vascular tone to norepinephrine therapy [20].

A further observation was the lowering of the heart rate after administration of Glibenclamide as well as a reduction in the CI demonstrated by the PiCCO^®^ measurements. Our observations suggest that this effect is not caused by myocardial damage but rather the return to normal circulatory values from an initially hyperdynamic condition. It seems as if parasympathetic autoregulation of the heart works negatively inotropic and chronotropic as the need for cardiac output has decreased. The fact that a reduction in HR was seen, reaching pre-CPB values 25 min after the application of Glibenclamide, and CI stayed significantly lower for the rest of the experiment suggests that these factors are independent and the lower CI is not caused by the lower stroke volume of the left ventricle.

Interestingly, lactate showed significantly higher levels in the SG at the end of the experiment. Due to the fact that there are no significant differences in urine output and CK-MB or Troponin levels between the groups, kidney or myocardial ischemia seems unlikely. Thus, we could exclude potential myocardial damage as the cause. Functional parameters such as the left ventricular contractility did not change significantly between the groups. An autopsy of the animals at the end of the experiment did not show any macroscopic signs of organ ischemia in any animal. One explanation for the increased lactate levels could be an interruption of mitochondrial respiration. This phenomenon is described in studies in which Glibenclamide led to a loss of cellular ATP and blocked the mitochondrial K_ATP_. This effect was concentration-dependent and was only observed after high-dose administrations [21,22]. Another explanation is that due to higher long-lasting SVRI, constriction of arteries and especially arterioles, there might be a reduced perfusion in the capillary bed leading to an increased anaerobe metabolism, especially in the skeletal muscles, skin, or intestines. Further studies are needed to examine this effect. Even though there were significant differences in hemodynamic measurements, it must be pointed out that the sample size of the animals examined is rather small. Further adverse effects may not be seen in small cohorts like this.

## 4. Materials and Methods

### 4.1. Porcine Model of Prolonged Cardiopulmonary Bypass

We established an animal model in adherence with European directive 2010/63/EU with a positive vote from the regional council of 6 June 2022. The study was reported in accordance with the ARRIVE guidelines [23]. In this porcine model, 25 female landrace pigs, 6 months old, with a body weight of 74.6 ± 1.5 kg, were examined. An initial pilot study to establish the experimental setup was performed on five animals. The remaining 20 pigs were divided into two groups with randomized adjudication of testing:

- control group using norepinephrine only (CG)
- study group using norepinephrine and Glibenclamide (SG)

The primary endpoints of this investigation were: dosages of norepinephrine, MAP, and SVRI.

### 4.2. Experimental Setup

After premedication, anesthesia was induced by intravenous bolus administration of 1 mg/kg propofol (Propofol, MCT Fresenius^®^, Fresenius Medical Care AG & KgaA, Bad Homburg, Germany), 3–5 µg/kg fentanyl (Fentanyl^®^, Piramal, Piramal Critical Care Deutschland GmbH, Hallbergmoos, Germany) and 0.1 mg/kg pancuronium (Pancuroniumbromid, Panpharma S.A., Luitré, France). Then, orotracheal intubation and ventilation were initiated, aiming at a tidal volume of 8 mL/kg body weight. Anesthesia was maintained through the continuous application of fentanyl and propofol. For basal volume substitution, a balanced full electrolyte solution (Sterofundin^®^ Iso, B Braun SE, Melsungen, Germany) ran continuously at a rate of 100 mL/h via an infusion pump. Continuous basic monitoring was initiated. A central venous catheter was introduced percutaneously into the external jugular vein for drug application, thermodilution, and CVP measurements. Invasive blood pressure and extended hemodynamic monitoring was established via the femoral artery using the PiCCO^®^ system (PiCCO^®^, Pulsion Medical Systems SE, Feldkirchen, Germany). A Foley catheter was introduced transurethral.

Surgery was performed under sterile conditions. After sternotomy, heparin (Heparin-Natrium, Braun SE, Melsungen, Germany) was administrated at a dose of 500 I.E./kg intravenously, aiming at an activated clotting time of >450 s. The ascending aorta and the right atrium were cannulated according to local standards. An aortic root vent was then placed for antegrade delivery of cardioplegia and venting of the aortic root. CPB was initiated and the extracorporeal blood flow was adjusted to maintain a (CI) of 2.4 L/min/m^2^, resulting in a MAP of 60–70 mmHg. Then, aortic cross-clamping (ACC) was performed and 1000 mL of Del Nido cardioplegic solution (4:1 crystalloid:blood; institutional pharmacy, Frankfurt am Main, Germany) was infused in a flow and pressure-controlled manner. Cardiac arrest was achieved under hypothermic conditions (35 °C). During ACC, lung ventilation was paused. After 60 min of ACC, another 500 mL of Del Nido cardioplegic solution was applicated. Blood gas and hemoglobin levels were measured online. Gas flow was adjusted to post-oxygenator CO_2_ measurements. After 60 min, rewarming was initiated to regain normothermia until the end of ACC. After 120 min of ACC, mixed venous reperfusion of warm blood (“hot shot”) for 3 min was carried out through the aortic root cannula, and then ACC was released. In case of ventricular fibrillation, defibrillation with internal paddles was performed. Ventilation of the lungs was commenced after the recruitment maneuver. After 60 min of reperfusion, the animals were weaned off CPB. In the next 90 min, post-CPB continuous hemodynamic monitoring was performed. This included MAP, PiCCO^®^ measurements, and the close documentation of required norepinephrine doses to keep the targeted mean arterial blood pressure.

In the SG, the study drug Glibenclamide (Glybenclamid G0639, Sigma-Aldrich, St. Louis, MO, USA) was administered 45 min after weaning from cardiopulmonary bypass (post-CPB) as bolus of 10 mg/kg at a rate of 500 mg/min. This bolus was directly followed by a continuous infusion via syringe pump in a dosage of 10 mg/kg/h for 45 min. Glibenclamide was dissolved with dimethyl sulfoxide (DMSO, D8418, Sigma-Aldrich). Hemodynamic effects of dimethyl sulfoxide without Glibenclamide were ruled out by administering it in four animals in CG and SG before sacrifice. A previous study had similar observations [18]. The experiment was finalized after 90 min of post-CPB. A total of 15 mL of T61 (tetracainhydrochlorid, membezoniumiodid, embutramid; Intervet Deutschland GmbH, Unterschleißheim, Germany) was applicated intravenously to sacrifice the animals.

### 4.3. Hemodynamic Data

**Figure 7:**
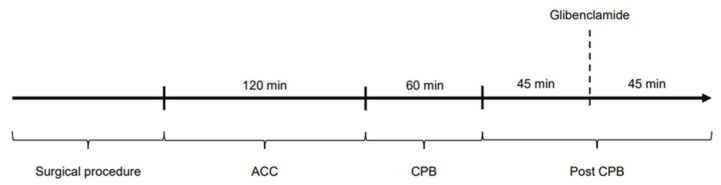
Experimental protocol of the study.

The study was divided into four time periods: Surgical procedure: time until the start of cardiopulmonary bypass and aortic cross-clamping; ACC: time on cardiopulmonary bypass while the aorta is cross-clamped (120 min); CPB: time on cardiopulmonary bypass during reperfusion (60 min); post-CPB: time after weaning from CPB, ongoing hemodynamic observations (90 min). Figure 7 shows the experimental protocol of the study. Hemodynamic parameters were documented every 15 min (ACC and CPB) and every 5 min in post-CPB, including HR, MAP, CI, SVRI, norepinephrine dose as well as blood gas parameters. Filling pressures and preload were kept within a standard range by administration of crystalloid solutions according to PiCCO^®^ measurements.

### 4.4. Laboratory Data

Blood samples were taken at baseline directly after the establishment of the central venous catheter and at the end of the experiment, shortly before sacrifice. Blood gas analysis, blood sugar, serum creatinine, lactate dehydrogenase, creatine kinase (CK and CK-MB), troponin-t, total protein, and albumin were measured directly. Tissue samples were taken from the lungs, heart, liver, kidney, femoral artery, aorta, and pulmonary artery. Blood samples: The concentration of the following parameters was determined using commercially available ELISA kits: Interleukin (IL)-1β, IL-6, IL-10 and TNF-α were measured by Quantikine^©^ porcine ELISA Kits (R&D Systems, Minneapolis, MN, USA). Concentrations of CD11b, PMN-elastase, malondialdehyde, eNOS, and iNOS were measured by Kits from BIOZOL (BIOZOL Diagnostica Vertrieb GmbH, Eching, Germany). Aortic tissue samples: ELISAs were carried out. iNOS and eNOS concentrations from aortic tissue were measured using Quantikine^®^ ELISA Immunoassay kits (R&D Systems, Minneapolis, MN, USA). Quantikine^®^ Elisa Immunoassay: The process of an ELISA is briefly explained using the R&D operating instructions [24].

### 4.5. Statistical Analysis

For an initial sample size calculation, we used an online sample size calculator (http://clincalc.com/Stats/SampleSize.aspx, accessed on 16 August 2022). We defined a minimally relevant group difference of 60%. With a power of 80%, accepting the probability of a type I error of 5%, 8 animals per group were required. As a safety margin, we aimed for 10 pigs/group. All the results were expressed as the mean ± standard error of the mean (SEM). The statistical software SPSS v29 (IBM SPSS Statistics, International Business Machines Corporation, New York, NY, USA) was used to analyze all the data. The data was tested for normal distribution using the Shapiro–Wilk test per measurement point and group. If the distribution was normal, the test for homogeneity of variance was then carried out using the Levene test. Within 10 min after Glibenclamide application, the Mann–Whitney *U* test was used to test for significance between CG and SG. Significant differences within one group were analyzed either using a paired-sample *t*-test for normally distributed data or a Wilcoxon matched-pair test. For all other time stamps, both groups were compared. With normal distribution and homogeneity of variance, the independent samples *t*-test was used. If the homogeneity of variance was absent, we used the Welch’s *t*-test. Otherwise, differences between the groups were shown using the Mann–Whitney *U* test. Body surface area (BSA) was calculated according to the following formula: BSA = 0.0798 × kg/body weight^2/3^ [25]. *p* values < 0.05 were considered significant.

### 4.6. Hematocrit (HCT) Correction

With the help of an HCT correction according to Schmid et al. [26], all blood parameters, which are given as concentrations, were multiplied by an HCT factor to reduce the effects of dilution. This factor was obtained by dividing the HCT before the start of the surgical procedure by the HCT at the end of the experiment.

## 5. Conclusions

In this porcine model, Glibenclamide administration led to a significant decrease in VS early after CPB during reperfusion by significantly raising MAP and SVRI, and reducing the need for norepinephrine. Therefore, Glibenclamide may have the potential to serve as an adjuvant therapy to attenuate vasoplegia during and after complex cardiac surgery. Further studies are needed to find the right dose of Glibenclamide that leads to a sufficient rise in MAP and SVRI with less adverse effects.

## Data Availability

The raw data supporting the conclusions of this article will be made available by the authors upon request.

## Abbreviations

The following abbreviations are used in this manuscript:

ACC	Aortic cross-clamping
ATP	Adenosine triphosphate
BSA	Body surface area
CG	Control group
CI	Cardiac index
CPB	Cardiopulmonary Bypass
CVP	Central venous pressure
HCT	Hematocrit
HR	Heartrate
K_ATP_	ATP-sensitive potassium channel
MAP	Mean arterial pressure
SG	Study group
SVRI	Systemic vascular resistance index
VS	Vasoplegic syndrome
